# The impact of the fipronil crisis on the financial performance of Dutch laying hen farms

**DOI:** 10.1186/s13071-020-04458-8

**Published:** 2020-11-23

**Authors:** Jaap Sok, Peter van Horne, Miranda Meuwissen

**Affiliations:** 1 Department of Social Sciences, Business Economics, Wageningen University, Wageningen, The Netherlands; 2 Subdivision Consumer and Chain, Wageningen Economic Research, Wageningen, The Netherlands

**Keywords:** Fipronil crisis, Financial performance, Financial distress, Partial budget, Laying hen, Family farm

## Abstract

**Background:**

Illegal use of fipronil as an insecticide in 2017 has caused substantial damage to Dutch laying hen farms. We assessed how the fipronil crisis has affected the financial performance of affected farms as well as unaffected farms. While affected farms faced culling their flocks and lost revenue, unaffected farms benefitted from temporary high egg prices.

**Methods:**

A three-step normative modelling approach is taken using financial statements and a partial budget. The estimations are for a 50,000 laying hen farm facing the fipronil crisis for 5 months. First, a baseline is created by generating an income statement of this laying hen farm representing a ‘normal year’. Second, incremental costs and revenue as a result of the fipronil crisis are estimated. Third, the baseline income statement is updated with the outcomes of the partial budget. This results in two additional income statements that report the net operating result of this farm being unaffected and affected by the fipronil crisis.

**Results:**

While in a normal year this average-sized farm has a net operating result of around 18,000 euros, profitability was estimated to be − 369,000 euros and + 169,000 euros for the affected and unaffected farm due to the crisis respectively. For affected farms, impacts were especially high as there was no government compensation or insurance.

**Conclusions:**

As Dutch farms typically operate as independent family farms, there was also no compensation from other chain actors. The affected farms therefore likely have faced financial distress and have had to increase debt or use their financial reserves for household consumption and restarting the business. Outcomes contribute to discussions around liability claims and cost-benefit assessments of measures to improve the chain food safety and rapid alert systems.
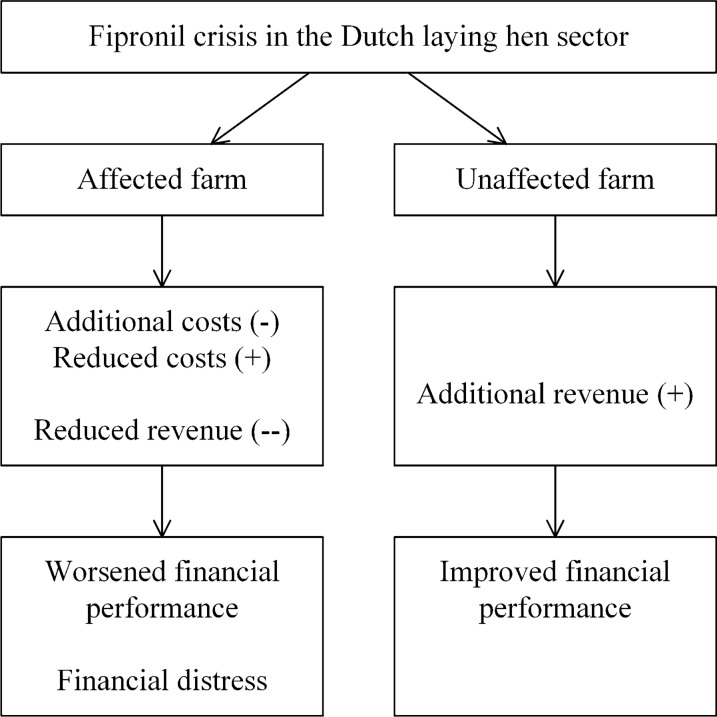

## Background

The use of a newly introduced insecticide to combat red mite led to a crisis in 2017 in the laying hen sector in The Netherlands after the insecticide was found to contain fipronil. Fipronil is allowed as an insecticide against lice for dogs and cats, but not for animals in food chains [1]. Public health risk was, according to the European Food Safety Authority, considered to be low; to exceed the level of toxicity (0.72 mg/kg), an adult (80 kg) would need to eat more than 17 jumbo eggs a day [2]. However, due to a large number of laying hen farms affected and the substantive amount of already exported eggs, the impact on the laying hen sector was large. More than 300 farms were temporarily shut down until it was proven that the stables were free from fipronil again, and more than 100 million eggs were discarded. Also, as forced moulting of laying hens appeared to be ineffective, more than 3 million chickens were culled. Even more, to regain consumer confidence in food safety, table eggs originating from The Netherlands, among others, were recalled in Germany.

The fipronil crisis did not only affect laying farms. The Dutch egg chain consists of several stages that work closely together [3]. Prior to laying hen farms, specialized breeding farms produce fertilized eggs; these eggs go to a hatchery, and the new-born chickens go to rearing farms. The young hens arrive at the laying hen farms a few weeks before the egg production cycle starts. Eggs are sorted and packaged by on-farm or specialized packing stations and then sold to various companies in retail, foodservice, processing (egg products), and export.

Concerning the farm-economic consequences of the fipronil crisis, losses for affected farms, i.e. farms on which the new insecticide was applied, differed from previous crises in the poultry sector such as outbreaks of epizootics and dioxin contaminations in poultry feed. Concerning the latter, poultry were not culled thereby leading to lower costs at the farm level [4]. In the case of epizootics, such as avian influenza, whole flocks are culled, but affected farms receive compensation from public-private animal health funds [5], often augmented with pay-outs from business interruption insurance to cover losses due to standstill [6]. During the fipronil crisis in The Netherlands, affected laying hen farms did not receive any indemnification. In contrast, unaffected farms benefitted from the fipronil crisis because of the temporary shortage of eggs and resulting high market prices.

In this article, our aim is to assess how the fipronil crisis has affected the financial performance of both affected and unaffected farms. Concerning affected farms, these insights are useful in the context of liability claims, for example. Outcomes also contribute to cost-benefit considerations that can further improve chain quality programs and alert systems. Moreover, benefits at unaffected farms are relevant in case of solidarity funds, as discussed in the aftermath of avian influenza outbreaks [6].

## Methods

### Modelling approach

**Fig. 1:**
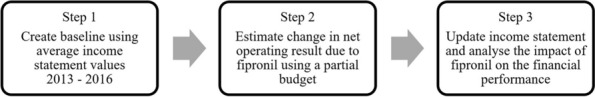
Three research steps to assess the impact of the fipronil crisis on the financial performance of an affected and unaffected farm

A normative modelling approach is taken, as laid out in Fig. 1, using financial statements and a partial budget. Farm-economic consequences are estimated in the context of the Dutch laying hen sector, which is dominated by family farms. The first step is to create a baseline by generating an income statement of a laying hen farm representing a ‘normal year’. The income statement is a ‘report of revenue and expenses ending with an estimate of net farm income’ and ‘provides an estimate of the value of products and services produced during an accounting period and the costs of the resources used to produce them’ [7].

We use a partial budget framework [7] in step 2 to estimate changes in farm income in a ‘normal’ year as a result of the fipronil crisis. We answer the following four questions:

1. What new or additional costs occurred [due to the Dutch fipronil contamination]?
2. What costs were reduced or eliminated [...]?
3. What new or additional revenues were received [...]?
4. What revenues were forgone [...]?

Partial budgeting is a form of marginal analysis as we assume that a fipronil contamination does not affect other decision-making aspects of the farm, such as the depreciation of a poultry house or the debt repayment schedule. The focus is on incremental costs and revenue. While in principle all four categories of the partial budget apply to both contamination states, for an unaffected laying hen farm only the category of additional revenue (question 3) is relevant. Outcomes of the partial budget model are used in step 3 and result in two updated income statements in addition to the baseline; one reports the income of an unaffected farm, the other of an affected farm. We analyse the impact of the fipronil crisis on financial performance using two indicators. First, we calculate the net operating result, which is ‘a criterion of profitability (also known in the sector as rentability) and thus indicates the remuneration for management and risk’ [8]. The net operating result represents the income available to provide a return to the production factors of capital (equity), labour, and management of the owner(s). Second, we calculate the earnings before interest, taxes, depreciation, and amortization (EBITDA). The latter is often used as a proxy for operating cash flow to measure the cash available to meet financial obligations [9].

### Data availability

Mainly two sources of data are used: farm accountancy data of Dutch laying hen farms publicly available from Wageningen Economic Research [10] via the Agro & Food portal (https://www.agrimatie.nl) and a commonly used reference guide (KWIN) that contains all sorts of quantitative base values that advisors, farmers, students, or researchers use to perform financial analyses [11]. The ‘Agrimatie’ database is mainly used to create the income statement baseline (step 1, Fig. 1, and Table 2 ‘normal year’). The KWIN guide is mainly used to calculate the different incremental cost and revenue factors in the partial budget model (step 2, Fig. 1, and Table 1). Some other references used for input values are a report that was prepared for the Dutch Ministry of Agriculture, Nature, and Food Quality by Horne et al. [12] and a report prepared by the Poultry Expertise Center (PEC), which is a public-private partnership in the Dutch poultry sector among companies, government bodies, and educational institutions [13].

**Table 1:** Partial budget over the period 31 July 2017–31 December 2017 of the impact of fipronil on the net operating result (50,000 laying hens)

	Unaffected farm	Affected farm
Additional costs	€ 0	€ 113,965
Fipronil manure disposal		€ 10,180
Poultry house cleaning		€ 10,000
Contaminated eggs disposal		€ 8056
Old flock disposal		€ 40,000
Old flock lump sum write-off		€ 45,729
Reduced costs	€ 0	€ 204,894
Feed		€ 200,099
Manure disposal		€ 4796
Additional revenue	€ 150,886	€ 0
Selling eggs	€ 150,886	
Reduced revenue	€ 0	€ 477,946
Selling eggs		€ 459,796
Slaughter value hens		€ 18,150
Change in net operating result	€ 150,886	€ −387,017

### Assumptions and delimitation

Economic consequences of a fipronil contamination depend on a range of factors that we cannot all take into account. Our perspective is the individual laying hen farm and not the egg supply chain. Most of our data consist of averages. We therefore work with a flock size of 50,000 hens [10]. We estimate incremental revenue and costs for the most adopted (60%) housing system in 2017, which is the ‘barn’, a floor housing system in which hens can move freely, as opposed to the cage system in which hens are confined in an ‘enriched cage’. In the barn system, hens have no outdoor access as opposed to the ‘free-range’ and ‘organic’ housing system [14].

**Fig. 2:**
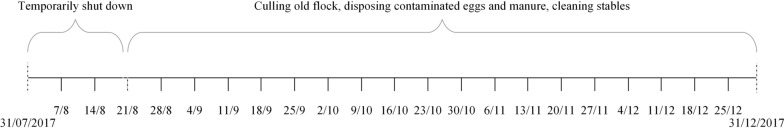
Illustration of a 5-month fipronil case: 3 weeks of temporarily shut down and 19 weeks of culling, disposing of eggs and manure, and cleaning

The income statements are made up for the year 2017, while for the partial budget we consider 5 months (Fig. 2). The first contaminated fipronil eggs were reported by the end of July in Belgium [15] and soon thereafter in The Netherlands. Farmers who treated their housing system with fipronil to control red mite infestation were temporarily shut down; consequently, no eggs, hens, or manure could leave the farm. Mainly two measures at the farm level were suggested to become free from fipronil: culling or forced moulting. The latter strategy is rational to take if the flock is still early in its egg production cycle. However, moulting often was not effective to get the hens free from fipronil making culling still needed [12]. Hence, we base incremental costs and revenue on a culling strategy.

Concerning depreciation of the flock, culling implies that the old flock is more quickly depreciated. The flock is assumed to have an egg production period of 68 weeks, starting 1 January 2017. For the treatment and reporting of depreciation, we follow Poppe [8] and regard depreciation as a fixed cost.

**Fig. 3:**
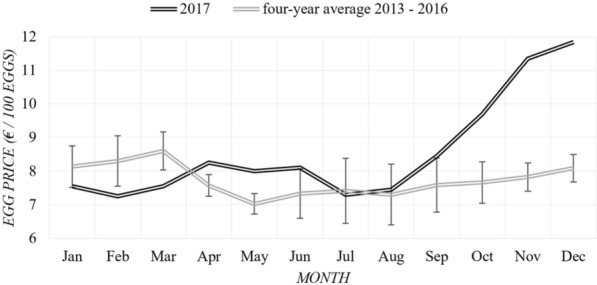
Egg price series monthly data from 2013–2017 [10]

Concerning egg prices, we use the average price over the first 6 months of 2017 (€ 7.8/100 eggs) and the average price of the last 4 months of 2017 (€ 10.3/100 eggs) to calculate additional and reduced revenue from selling eggs for the farm being affected and unaffected. Figure 3 provides an overview of monthly egg prices from 2013–2017, the first 4 years as monthly averages. In the first quarter, the monthly egg prices in 2017 were below the 4-year average (2013–2016), while in the second quarter this was the opposite. However, since we express the impact on the operating result in a year, the monthly variation is less important for our calculations. Based on the egg price series data, a price level of € 7.8/100 eggs well represents the price level in a ‘normal year’.

Additional file 1 can be accessed to examine the input values and verify the calculations made for the partial budget and income statements.

## Results

We start by presenting the partial budget results for the 5 months as laid out in Table 1. An unaffected laying hen farm operating under ‘normal’ circumstances in 2017 had no additional or reduced costs. The only way fipronil impacted the financial performance is via the revenue side: these farmers received over the last 5 months of 2017 a substantially higher price for their eggs (on average € 2.6/100 eggs). The change in net operating result for an unaffected laying hen farm is estimated at € 150,886, which is about 8.5 times the net operating result in a ‘normal year’.

In contrast, an affected laying hen farm had considerable additional costs. About 40% of these costs relate to depreciation, more specifically the lump sum write-off of culled hens. Instead of 52 weeks of depreciation in a normal year, the full useful life (production cycle) of 68 weeks had to be depreciated. The other 60% entailed specific costs made for the cleaning of the poultry house and the disposal of hens, eggs, and manure. However, there were fewer additional costs than reduced costs. With the culling of the flock, feed was saved while less manure had to be disposed of. The biggest cause for the negative change is at the revenue side, i.e. the production standstill of about 5 months causes an estimated drop in revenue of € 477,946. A small fraction of the reduced revenue is due to the slaughter value forgone as the hens had to be culled and disposed of. The change in net operating result for an affected laying hen farm is estimated at € − 387,017.

At this point, we would like to emphasize the impact of depreciation costs on the net operating result. We consider only changes in revenue and costs in the year 2017. In our calculations, only 16 weeks of depreciation had to be written off extra for an affected farm, as we assumed the flock to have an egg production period of 68 weeks, starting as of January 1. Suppose the flock were younger, thus earlier in its production cycle at the start of the shut-down period (Fig. 2); then the old flock lump sum write-off would have been much higher. Costs of depreciation increase with € 2858 per week.

Table 2 presents the income statement over the whole year of 2017 in which we report the net operating result estimation of a laying hen farm in a ‘normal’ year. The net operating result we estimate in a normal year is € 17,729. One should note that there have been quite some fluctuations in the annual net operating result figures over the last years in the laying hen sector including €-112,600 in 2013, € 20,200 in 2014, € 130,200 in 2015, and € 101,100 in 2016 [10]. Note also that the average of the aforementioned income figures slightly differs from our estimation because of a different income format used to calculate depreciation of the flock.

**Table 2:** Income statement over 2017 of a laying hen farm in a ‘normal year’, in a fipronil unaffected and affected state (50,000 laying hens)

	Normal year (baseline)	Unaffected farm	Affected farm
Revenue			
Turnover (eggs)	€ 987,350	€ 1,138,236	€ 527,554
Other returns (meat, other business activities)	€ 94,600	€ 94,600	€ 94,600
Total revenue	€ 1,081,950	€ 1,232,836	€ 622,154
Variable costs			
Feed	€ 596,250	€ 596,250	€ 396,152
Manure disposal	€ 9625	€ 9625	€ 15,010
Other allocated costs (e.g. maintenance)	€ 79,600	€ 79,600	€ 79,600
Poultry house cleaning			€ 10,000
Disposal contaminated eggs			€ 8056
Disposal costs old flock			€ 40,000
Total allocated costs	€ 685,475	€ 685,475	€ 548,817
Gross margin	€ 396,475	€ 547,361	€ 73,338
Fixed costs			
Depreciation of flock	€ 148,621	€ 148,621	€ 212,500
Other non-allocated costs	€ 230,125	€ 230,125	€ 230,125
Total fixed costs	€ 378,746	€ 378,746	€ 442,625
Estimation of the profitability			
Net operating result	€ 17,729	€ 168,615	€ −369,287
Estimation of the repayment capacity			
EBITDA (excl. depr. flock)	€ 155,154	€ 306,040	€ −231,862

The outcomes of the partial budget are inserted into the income statement figures, allowing to calculate the net operating result for a laying hen farm being unaffected or affected by the fipronil crisis. Due to the higher selling prices, the returns for an unaffected farm increased by more than 15% compared to the returns in a normal year. Since the cost structure is not impacted, the net operating result is more than nine times higher, while the EBITDA almost doubled.

For an affected farm, both revenue and costs changed considerably. The turnover from selling eggs for an affected farm decreased by more than 40%. Allocated costs initially increased because of the additional costs of the cleaning of the poultry house and the disposal of hens, eggs, and manure. But the reduced cost factor of feed of € 200,099 resulted in an overall decrease in the allocated costs of € 136,658 compared to the allocated costs in a normal year.

The gross margin (revenue minus variable costs) is not large enough to cover fixed costs. The latter increased substantially for an affected farm as the fipronil contaminated flock was culled, and the remaining book value and anticipated slaughter (salvage) value had to be written off as a lump sum. The net operating result of an affected laying hen farm is estimated at € –369,287 and the EBITDA at € –231,862.

The profitability calculated under a normal year is too low to provide satisfactory returns to the unpaid production factors of the owner’s capital, management, and labour. The average equity of a Dutch laying hen farm over 2013–2016 was € 743,175 [10]. A net operating result of € 17,729 is not enough to provide remuneration for this amount of capital as well as for the labour hours spent of the farmer. However, due to the fipronil crisis, unaffected farms had a profitable year with decent returns, increasing the owner’s equity and working capital. Affected farms, on the other hand, most likely had to increase debt or use their financial reserves to counterbalance the negative income and make a continuation of the business possible and restart egg production with a new flock. The EBITDA estimation indicates that these farms have likely experienced financial distress after the fipronil crisis.

## Discussion and conclusions

Illegal use of fipronil as an insecticide in 2017 has caused substantial damage to Dutch laying hen farms. In this article, our aim was to assess how the fipronil crisis has affected the financial performance of both affected and unaffected farms. While affected farms faced culling their flock and lost revenue, unaffected farms benefitted from temporary high egg prices. Note, however, that unaffected farms selling eggs via contracts did not benefit. Estimations are for a 50,000 laying hen farm facing the fipronil crisis for 5 months. While in a normal year this farm has a net operating result of around 18,000 euros, profitability was estimated to be − 369,000 euros and + 169,000 euros for the affected and unaffected farm because of the crisis respectively. For affected farms, impacts were especially high as there was no government compensation or insurance. As Dutch farms typically operate as independent family farms there was also no compensation from other chain actors. The affected farms therefore likely faced financial distress and had to increase debt or use their financial reserves for household consumption and restarting the business.

A normative, deterministic modelling approach was taken to assess the financial performance of fipronil affected and unaffected farms. We estimated for these two farm types changes in revenue and costs based on 50,000 laying hens, which represents an average farm size. The Dutch laying hen sector in 2017 consisted of 860 farms [3], which vary in terms of size, capital structure, technical performance, innovativeness, etc. These and other characteristics all influence financial performance and the impact of a shock such as the fipronil affair. For example, large and modern farms, especially those that are financed mainly by outside capital, are committed to proportionally more depreciation and interest costs. A positive, empirical modelling approach to address the impact of these characteristics was not possible to apply; from the farm accountancy data sample, affected farms could not be distinguished from unaffected farms.

Nevertheless, estimations of the impact of the fipronil crisis on the financial performance of Dutch laying hen farms are still conservative as we only accounted for direct and visible farm-economic (monetary) consequences in 2017. At the farm level, the financial performance in (the beginning of) 2018 most likely still deviated positively from a ‘normal year’ for unaffected farms and negatively for affected farms. At the sector level, the financial performance of laying hen farms has been affected by efforts needed to retrieve export markets and to regain consumer trust, see e.g. [16]. Also, costs of monitoring and disruptions along the value chain were not included.

We further stress that quantifying the change in revenue and costs does not provide the full story of how the fipronil crisis has affected laying hen farm owners. The Dutch laying hen sector is dominated by family farms. Our financial analysis did not consider qualitative (non-monetary) factors, such as the farm household’s feelings of shame and anger, see e.g. [17]. Reciprocal relationships exist between financial distress and psychological distress [18].

Outcomes contribute to discussions around liability claims and to cost-benefit considerations for further improving chain quality programs and alert systems. Moreover, benefits at unaffected farms are relevant in case of solidarity funds, as discussed in the aftermath of avian influenza outbreaks [6].

## Supplementary information

**Additional file 1.** Fipronil input values and calculations.

## Data Availability

All data generated or analysed during this study are included in this published article [and its supplementary information files].
